# Healing Through Humanized Care: Lessons from a Patient-Centered Perinatal Loss Protocol

**DOI:** 10.3390/healthcare13030242

**Published:** 2025-01-24

**Authors:** Rosana Caro-Costa, José Manuel Alcaide-Leyva, Lourdes García-Luque, Sandra Alcaraz-Clariana, José Miguel Guzmán-García, Maria del Rocío Jiménez-Mérida

**Affiliations:** 1 Hospital Universitario Reina Sofía, 14004 Córdoba, Spain; rosanacarocosta@gmail.com; 2 GA16—Nutrition, Dietetics, and Healthy Lifestyle Habits Research Group, IMIBIC (Maimonides Institute for Biomedical Research of Córdoba), 14004 Córdoba, Spain; z92guzgj@uco.es (J.M.G.-G.);; 3 Department of Nursing, Pharmacology, and Physiotherapy, University of Córdoba, 14004 Córdoba, Spainm72alcls@uco.es (S.A.-C.)

**Keywords:** perinatal loss, bereavement care, nursing staff, emotional support

## Abstract

**Background/Objectives:** Perinatal loss is a challenging experience that significantly impacts families and healthcare professionals. This study aimed to explore the perceptions and experiences of the nursing team in the gynecology unit at a Spanish hospital regarding the implementation of a new protocol for perinatal bereavement care. **Methods**: A qualitative descriptive study was conducted involving a focal group interview with nursing staff from the gynecology unit. Participants were selected through purposive sampling, and data were analyzed using thematic analysis to identify recurring patterns and insights. **Results**: The findings revealed a disparity in attitudes toward perinatal bereavement care, with some nurses demonstrating empathy and understanding, while others expressed discomfort and avoidance behaviors. Barriers to effective care included a lack of formal training, insufficient emotional support, and practical challenges in implementing the protocol. Participants suggested improvements such as enhanced communication training, the appointment of a bereavement coordinator, and earlier psychological interventions to support both families and staff. **Conclusions**: The study highlights the need for continuous education and emotional support to facilitate the adoption of perinatal bereavement protocols. Involving nursing staff in protocol development and addressing systemic barriers can improve the quality of care for bereaved families while supporting healthcare professionals’ emotional well-being.

## 1. Introduction

### 1.1. Understanding Perinatal Bereavement

Perinatal bereavement represents one of the most challenging and traumatic experiences for families, profoundly impacting their psychological, physical, and social health. The World Health Organization defines perinatal death as occurring between 28 weeks of gestation and the first seven days of life [1]. However, in many contexts, including Spain, this definition extends to losses occurring from the earliest stages of pregnancy up to the first month of the baby’s life [2]. In Spain, the perinatal mortality rate was 4.03 per 1000 live births in 2021, translating to approximately 1450 perinatal losses during that period [3].

The impact of perinatal loss transcends emotional dimensions, also affecting the physical health of parents and their family dynamics. Consequences include symptoms such as anxiety, depression, and post-traumatic stress disorder, alongside physical issues like appetite loss, insomnia, and chronic illnesses. These losses can also strain relationships, increasing conflict frequency and emotional isolation within couples [4]. In Spain, the cultural stigma surrounding death further complicates the grieving process, limiting open discussions and affecting how families cope with their emotions or grief [5]. In this context, nursing professionals play a critical role in mitigating the pain of affected families. Interventions such as post-mortem contact with the baby, the creation of tangible memories (memory boxes), and the use of devices like CuddleCots have proven effective in facilitating the grieving process [6]. However, these interventions require healthcare professionals to have appropriate training and resources to provide care tailored to the needs of patients and their families [7].

In response to these challenges, a structured perinatal bereavement protocol was recently implemented at Hospital Universitario Reina Sofía. This protocol aims to standardize care practices and provide comprehensive emotional support to grieving families. Key components include the use of the CuddleCot to allow families extended time with their baby, memory boxes with personal keepsakes, and immediate psychological support services. The protocol was developed based on international best practices and adapted to the hospital’s cultural and clinical context. However, its successful implementation relies heavily on staff acceptance and proper training, making it essential to explore the nursing staff’s perceptions and experiences regarding its application.

Globally, there is an increasing recognition of the need for structured protocols to address perinatal bereavement. Countries such as the United Kingdom and Australia have implemented comprehensive guidelines that emphasize family involvement and the creation of supportive environments [8,9]. These protocols highlight the value of providing tangible memories and ensuring privacy and dignity during the grieving process, reinforcing the importance of patient-centered approaches.

### 1.2. The Relevance of Patient-Centered Care

Patient-centered care (PCC) prioritizes the preferences, values, and needs of those being cared for, promoting their active participation in the care process. This model is particularly critical in perinatal bereavement, where families often face complex emotional and cultural challenges. PCC ensures that families feel acknowledged and respected throughout the grieving process, addressing not only clinical needs but also emotional and cultural dimensions [10]. Moreover, it supports healthcare professionals by offering structured frameworks to navigate emotionally demanding situations. By fostering respect, empathy, and holistic care, PCC provides a foundation for healthcare professionals to navigate emotionally demanding situations while improving outcomes for patients.

Perinatal bereavement protocols have recently been introduced in Spain to improve the experience of affected families. These protocols includes strategies such as the use of CuddleCots, family accompaniment during grief, and the provision of psychological support [11]. While these initiatives align with the principles of PCC, they also highlight gaps in training and institutional support that need to be addressed to maximize their impact [12].

### 1.3. The Role of Nursing Professionals

Nursing professionals, as frontline caregivers, play a pivotal role in implementing perinatal bereavement protocols. However, they often face significant challenges, including limited training, insufficient emotional support, and a lack of clear guidelines.

In Spain, this is further complicated by the cultural taboo surrounding death, which creates barriers for both families and healthcare staff in openly addressing grief [5]. Prior to the implementation of new protocols, practices were inconsistent. Deceased babies were transferred directly to the mortuary while mothers were relocated to other wards, often without the opportunity for meaningful closure. The introduction of initiatives like CuddleCots and memory envelopes aimed to address these gaps by fostering postmortem contact and providing tangible keepsakes to support grieving families [11,13].

Despite these efforts, the protocol’s success depends heavily on its alignment with the cultural context and the readiness of staff to embrace its principles. For example, some nursing staff expressed discomfort with certain practices, such as prolonged stays with the deceased baby, highlighting a need for enhanced emotional support and training for both staff and families. Understanding these dynamics is critical not only for optimizing care but also for addressing systemic barriers that hinder compassionate practices [14].

This study seeks to explore the perceptions and experiences of the nursing staff regarding the implementation of the new perinatal bereavement protocol and to identify areas for improvement to enhance family-centered care.

## 2. Materials and Methods

### 2.1. Qualitative Approach and Research Paradigm

This study employed a descriptive approach to explore the experiences of nursing staff with the implementation of the new perinatal bereavement protocol at a hospital in southern Spain. This design is particularly suited to exploring emotional and subjective experiences, enabling a nuanced understanding of how nursing staff navigate the challenges of perinatal bereavement care [15]. The descriptive design facilitated a comprehensive and authentic understanding of the participants’ lived experiences, helping to capture their thoughts and perceptions of care in perinatal loss situations as they were expressed [16].

### 2.2. Context

The study was conducted in the gynecology unit, where a new perinatal bereavement protocol was recently implemented. The protocol was implemented 6 months before the data collection. It included an algorithm of action, as well as the professionals in charge of each task. It also added the possibility, at the request of the families, of using the CuddleCot or creating a baby memory box. In addition, families were given the option to talk to a psychologist to better cope with the bereavement. Prior to the protocol’s introduction, bereavement care at this unit lacked structure, often leaving families without adequate opportunities for closure [13].

### 2.3. Sampling Strategy

Participants were selected using non-probabilistic convenience sampling, ensuring accessibility while aiming for discourse saturation. The inclusion criteria required participants to be registered nurses or auxiliary nurses working in the gynecology unit with at least six months of experience in perinatal bereavement care. Exclusion criteria included nursing staff with no direct involvement in bereavement cases. Since the protocol was introduced recently, the number of cases available for analysis is inherently limited. Furthermore, the annual incidence of perinatal deaths is relatively low, further restricting the pool of potential participants. These circumstances justify the selection of a small sample size. Data saturation was considered reached when no new themes emerged during the interviews, and participant contributions began to overlap significantly [17]. For this study, only 4 participants were enough for data saturation.

### 2.4. Ethical Issues Pertaining to Human Subjects

The study adhered to ethical principles outlined in the Declaration of Helsinki [18]. Approval was obtained by the Institutional Review Board of Hospital Universitario Reina Sofía (Comité de Ética de la Investigación Provincial de Córdoba) on 30 January 2024 (Acta no. 357). The consent form for participation was distributed to all participants and signed. Measures were implemented to ensure data confidentiality, including anonymization of transcripts and secure storage of data. To avoid any emotional distress, several measures were implemented to minimize emotional risks to participants, particularly given the sensitive nature of the topic. Firstly, participants were provided with detailed information about the study’s objectives, procedures, and potential risks before giving their written informed consent. They were informed of their right to withdraw at any time without any consequences. Data collection was conducted in a familiar and comfortable setting to foster a sense of safety and openness. A trained moderator facilitated discussions with sensitivity, ensuring that participants felt heard and respected throughout the process.

### 2.5. Data Collection Methods

The group interview was conducted as focus group in the gynecology ward’s nursing station. This setting was chosen for its familiarity to participants, reducing potential anxiety and encouraging openness [19]. The interview guide included broad, open-ended questions designed to explore participants’ perceptions of the protocol, its strengths, weaknesses, and suggestions for improvement. Audio recordings were made with participants’ consent, and transcripts were anonymized during the data processing phase.

### 2.6. Data Processing and Analysis

The main researcher was responsible for transcribing the interviews and coding the participants. For this reason, the sample could not be blinded for all researchers. After the coding, the interviews were anonymized and transcribed and shared in a password-protected cloud folder accessible only to the research team.

Interview data were analyzed using a descriptive approach [20], through inductive coding, allowing categories to emerge naturally, with the researcher acting as a mediator who organizes and synthesizes the information without imposing a rigid theoretical framework. This approach is particularly valuable when the goal is to provide a detailed and accessible understanding of the phenomenon under study. The NVivo program (Version 15, Lumivero, Denver, CO, USA) was used for the analysis of findings [21].

### 2.7. Techniques to Enhance Trustworthiness

To ensure the rigor and quality of the qualitative part, the criteria proposed by Lincoln and Guba were followed, including methodological appropriateness, data triangulation, and reflexivity [21]. The researchers’ prior professional experience in the gynecology unit was acknowledged as a factor influencing reflexivity and the interpretation of findings. To enhance objectivity, data collection was conducted using open-ended questions and a semi-structured approach to minimize leading responses. Two researchers independently coded the data and compared results to identify and address potential bias in analysis. Lastly, member checking was performed by sharing preliminary findings with participants to confirm that the interpretations accurately represented their experiences.

## 3. Results

A group interview (focus group) was carried out with the participation of four members of the nursing staff. Among the characteristics of the participants, all four were women, two nurses and two nursing assistants, between the ages of 50 and 63, two married, one divorced, and one single. The professional experience in the gynecology unit varied; the longest was 14 years, and the shortest 3 years in that service (Supplementary Materials).

### 3.1. Experiences of Nursing Staff

The experiences of the nursing staff were generally negative. The incorporation of the new protocol has meant a paradigm shift in the care of families after perinatal loss compared to the care provided under the old procedure. Its implementation was met with diverse reactions from nursing staff. Their experiences revealed a mix of emotional, professional, and practical challenges, as well as areas of strength.

○ Strengths
  ▪ Empathy and personalization in care

The nursing staff is the most direct attendant in situations of perinatal loss, so it is of great importance to show empathy towards the family, carrying out actions that ensure dignity and respect for the family’s grief. “Since the mother was going to hold her baby, I tried to prepare everything as best as I could so that she wouldn’t have that memory engraved in her mind” (P1). Participants carried out actions to reduce the family’s pain and to help them accept the situation, such as identifying the baby’s name or preparing the baby’s body before the family met them.

○ Weaknesses
  ▪ Knowledge for understanding

Both nurses and nursing assistants should be trained to assist in situations of perinatal loss in order to offer adequate support to the family, although few nurses had this type of training. “I don’t have any information, nor have we been given it here. I don’t have much training either… I think that’s what it feels like” (P2). The nurses claimed that they are not trained to attend to a family who has lost their baby, so they learn as they go along with their own experiences. Due to this poor training, the nursing staff reflects avoidance at the time of performing the necessary care, both to the family and to the baby. “It’s a situation that you try to avoid entering because sometimes you don’t know how to face or how to console the family. Well, at the beginning a bit more… Then you get the hang of it” (P1).

  ▪ Empathizing—not always an easy task

In contrast to the empathetic attitude that they show at first, and especially in bereavement situations, attitudes of lack of empathy appear along the process of care. “Here everyone (the family) comes in to say goodbye to the child, so this is not fair for us” (P2; all assent). Some participants expressed difficulty in empathizing with the personal characteristics and life experiences of the patients, like not being a mother. “Not having been a mother myself, maybe I empathise less” (P3). Some examples they provided were the staff’s discomfort by the flexibility of the family visitation rules, or the comparative grievance with the farewell after the death of an older child, “If a child dies at the age of 8 (…), you are not allowed to keep him in your arms for 6 h (…) Because this mother, what bond does she have with this child if she has just seen him?” (P4)

  ▪ Emotional imprint

Participants expressed difficulty in coping with the sight and touch of the baby in the cadaveric state, making the caregiving experience unpleasant. “I used to sleep and dream about the child (…) when I see a situation like that with that child there, lifeless, it is a deep pain for me” (P3). Moreover, they expressed difficulty trying to disconnect outside of work after assisting the baby.

  ▪ Rejection for self-protection

Due to the difficulties of bereavement care in a perinatal loss, already mentioned above (emotional consequences, lack of knowledge…), the nursing staff reflected rejection at the time of performing the necessary care. “I found the experience the most unpleasant thing in the whole world” (P1).

### 3.2. The Perinatal Bereavement Protocol

○ Positive key points

The nursing staff highlights some positive features of the current protocol. Some of them are the creation of the memory envelopes and the flexibility in the accompaniment of family members. “Normally the midwives come up with the memory envelope, (…) a bracelet identifying the baby, the footprint, if they have put a hat on the baby… They leave it there in a little envelope so that they can keep it as a remembrance” (P1). Another major consideration of the protocol is the adaptation of the environment, not admitting mothers to the maternity ward. “As bad as it is… you can’t even leave it on the 3rd floor (delivery ward), they’re already there listening to other mothers with their babies crying, and obviously they’re not going to take it up to the 4th floor (maternity ward)” (P4; all assent).

Finally, participants highlighted the psychological care service available to mothers and families to help them during the mourning process. These strategies aimed to reduce the family’s pain and help them grieve. “It is true that when women are given information about the psychological support consultation, it helps them in the grieving process (…) (P2).

○ Negative key points

According to the nurses and nursing assistants interviewed, the protocol was implemented without knowledge on their part, in addition to the gynecologists’ lack of information. “You are here alone at night shift and suddenly they call you and you don’t know where you stand because you don’t even have anyone to ask” (P4; all assent). “The gynaecologists don’t know where to call, nor do you” (P3).

They also note a poor way of transmitting information on the protocol, by an online training. Participants thought that was not the best way of training the staff. “A circuit (online training) was done, but I don’t think that is the way to transmit information to a staff that takes care of this kind of patient” (P1). As opposed to her peers, one of the nursing assistants believed that training was not necessary in this regard, that the support offered to the family depends on personal sensitivity.

Moreover, the nursing staff emphasize the lack of knowledge that parents have regarding the physical appearance of their babies. “I think that the mother and father should be told what the child will look like, so that they too can get the idea that this is not a body like that of an adult person, which, when it dies, has its own consistency for a few hours. I think they should prepare the mother and the father so that they know how they are going to find their child so that they can keep it for 6 h or say ‘well, we’ll see the baby 5 min’” (P2). From their point of view, this is an emotional shock and can impair the bereavement process.

○ The emotional bereavement of the families

According to the perspective of the personnel who assist families who have suffered a perinatal loss, mothers and fathers who have the baby with them are in a worse state of mind than the others that do not have the baby in the room. “I think that the families who have the baby here are more broken when they take the baby away, it’s harder for them to finish pushing (…) (P2; other participants assent). “But now that the foetus is also here, that’s negative (…) I imagine that the mother is already completely broken” (P3).

  ▪ Protocol implementation

Regarding the characteristics of the protocol itself, there is clear opposition in many aspects from the nursing staff interviewed. The implementation of the protocol lacks good management, since the bureaucratic and administrative part is not as agile as the process needs to be. The nurses confirm the poor management of the cases of bereavement process. “Very poorly assessed. In other words, the experience may be good, but here it was implemented a little too fast”(P4; other participants assent).

In the first place, they do not believe that the CuddleCot is adequate for this type of situation, because the families prefer to keep the baby in their arms before placing it in the crib, so the purpose of keeping the fetus in better conditions is not achieved. “The cradle of hugs is a fallacy, because the cradle of hugs is a metaphorical and poetic name that they wanted to give to something” (P2). “When a woman comes with a baby that has died, it is passed from hand to hand by all the relatives. The tendency is to hold it and not to use it for anything” (P4; all assent).In addition, there is also disagreement regarding the transportation of the baby to the mortuary, since it is done with the same stretcher as for adults, which makes it much more difficult to contain the baby and produces a greater emotional impact.

### 3.3. The Perinatal Bereavement Protocol—What Comes Next?

As for tools to improve the protocol and assistance in bereavement situations, the nursing staff suggests making changes related to transportation to the mortuary adapted to the circumstances of the corpse, emotional assessment of the families afterwards to check whether the protocol has had a positive effect on their bereavement process, offering greater psychological support to parents, and eliminating the CuddleCot due to its uselessness. “They should assess whether what they are projecting, that these women should be followed up, that these patients should be interviewed over time to see if being accompanied by their baby in the room for the hours that it was there has helped them, in their process of grief, of mourning, has helped them in any way (…) I would follow them up over time to see if it has helped them in any way” (P1). “I would remove the cradle, I would put in the child transport” (P2; other participants assent). “I think that maybe more help in the form of a psychologist, from the first moment, not waiting for a week, but from the first moment (…)” (P3; other participants assent).

## 4. Discussion

This study focused on the vision and experiences of the nursing team of a gynecology unit regarding the new protocol for perinatal loss. Considering the attitudes of nurses towards perinatal bereavement care, one interviewee demonstrated attitudes consistent with empathy and understanding, whereas the others displayed negative attitudes towards perinatal bereavement. This disparity highlights the complexity of emotional engagement in bereavement care, often influenced by variables such as professional experience, age, and personal resilience. Scientific evidence reflects that these differences may stem from the characteristics of professionals caring for bereaved families, such as age, experience, or bereavement training [22]. Additionally, some studies emphasize that expressing emotions is often devalued due to its association with femininity, being relegated to a lower priority compared to cognitive or technical skills [23]. This stigma surrounding emotional expression may prevent nursing staff from providing meaningful emotional support, contributing to personal distress and avoidance behaviors [24].

To address these barriers, integrating cultural sensitivity into training programs is essential. Simulation-based learning and role-playing exercises can immerse healthcare professionals in culturally relevant scenarios, allowing them to practice navigating delicate conversations and addressing cultural taboos effectively [25]. For instance, tailored workshops that reflect local customs regarding perinatal loss could help bridge gaps in understanding between families and healthcare providers. Furthermore, the inclusion of culturally competent communication strategies can promote empathy and reduce misunderstandings during highly emotional situations [26].

The difficulties encountered in caring for bereaved families encourage avoidance behaviors [27], as seen in our findings, and nurses and midwives experience emotional exhaustion when they become emotionally involved with parents [28]. Nurses require a caring organizational culture that emphasizes holistic care, developing and strengthening skills to connect with parents and manage the emotional demands of this care [29]. Structured debriefing sessions and reflective practices after perinatal loss cases can help mitigate emotional exhaustion and foster resilience among nursing staff [30]. Reflective practices, in particular, have been shown to improve coping strategies and reduce burnout among healthcare providers [31].

Emotional support should also be incorporated into training programs through strategies such as mindfulness practices, resilience workshops, and stress management sessions tailored to the needs of nursing staff. These interventions can empower healthcare providers to address their own emotional well-being, thereby improving their capacity to care for bereaved families [32]. Research highlights the success of such initiatives in reducing emotional distress among healthcare workers and fostering long-term resilience [33].

Furthermore, evidence supports that caring for families experiencing perinatal loss impacts nursing staff psychologically and emotionally, although no feelings of fear or rejection were reported in this study, differing from previous research findings [34]. This cultural difference may stem from varying coping mechanisms or differences in exposure to bereavement training [35]. Personal coping strategies, as reflected in our study, appear to play a key role in protecting nurses’ mental health during these experiences [29]. This highlights the need for tailored support programs that focus on enhancing emotional resilience among nursing professionals.

Internationally, programs in the UK and Australia have demonstrated the effectiveness of integrated approaches to bereavement care training, combining cultural sensitivity and emotional support [33,36]. These programs emphasize the importance of a multidisciplinary approach, involving psychologists, cultural advisors, and experienced nursing staff to deliver comprehensive training. Evidence suggests that such strategies not only improve patient outcomes but also enhance staff confidence and job satisfaction [37].

Additionally, there is a general lack of knowledge and sufficient skills among healthcare professionals regarding perinatal loss, as highlighted by our participants. Key factors in effectively supporting women who have experienced perinatal loss include attitude, communication skills, and specific bereavement training.

However, these skills can be difficult to develop due to the interactive nature of the grieving process, requiring a solid foundation in communication beforehand [38]. As seen in our findings, nurses often learn to manage such situations through professional experience or peer support [39,40]. Recent studies emphasize the importance of immersive training programs incorporating simulations and role-play, which have demonstrated improvements in nurses’ confidence and communication skills during end-of-life care [41,42]. Moreover, attending educational programs on perinatal loss significantly increases nurses’ knowledge, confidence, and comfort levels when providing bereavement care [43].

Regarding the protocol itself, various perinatal bereavement protocols exist globally, each adapted to local sociocultural aspects. Some studies report that staff may find such protocols unsettling or morbid, which hinders their application. To overcome these barriers, it is crucial to design training programs that incorporate cultural competence and provide psychological support to healthcare professionals [44]. This approach helps staff to understand the significance of culturally sensitive practices and manage their own emotional responses. These limitations can be addressed by appointing a reference professional trained in perinatal loss management, who can guide the staff in incorporating these protocols into practice [45]. The absence of such a figure, as revealed in our findings, emphasizes the need for a bereavement care coordinator who can provide ongoing support, education, and reassurance to the nursing team [46,47]. Coordinators have been shown to reduce staff distress and improve compliance with bereavement care protocols [48].

Another negative aspect raised by participants was the experience of the parents, who were perceived as having worsened emotional states, contrary to what the evidence suggests. Accompanying the mother while holding the deceased baby has been shown to positively impact long-term emotional outcomes, facilitating the grieving process [49]. This contrast may be linked to insufficient family guidance and the lack of staff training to support these interactions. Implementing structured guidelines on how to prepare families for these moments could significantly enhance their grieving process [44]. Evidence shows that well-trained staff can facilitate these moments to enhance family well-being.

Various strategies exist to support families experiencing perinatal loss, such as memory boxes containing meaningful items, clay impressions of the baby, photography, parent-based support groups, and intergenerational family support. Additionally, training healthcare personnel who will care for the mother and baby is essential. Our participants suggested complementary strategies, including continuous evaluation of family satisfaction with the protocol, improving transportation processes for the baby to the mortuary, and initiating psychological intervention earlier in the process. Recent studies also highlight the value of involving families in the development of support measures, ensuring that these resources are both culturally appropriate and emotionally supportive [50].

The findings of this study reveal critical challenges in the implementation of the perinatal bereavement protocol, including gaps in staff training, emotional strain on healthcare professionals, and logistical difficulties. To address these issues, several actionable improvements are recommended: revising the use of the CuddleCot based on family preferences, implementing early psychological interventions, strengthening staff training through simulation-based learning, improving protocol communication among healthcare staff, and adapting procedures for the respectful transfer of deceased infants. Implementing these improvements, as suggested by the nursing staff, could significantly enhance the protocol’s effectiveness and ensure more compassionate, family-centered care.

In conclusion, it is essential that staff involved in protocol implementation participate in its development, as they have the clearest understanding of patient needs. Collaborative development processes, involving multidisciplinary teams, ensure that protocols are realistic, effective, and well-received [51]. Failure to include nursing staff can lead to suboptimal care and interference in the grieving process. Furthermore, healthcare institutions must prioritize ongoing training for their staff, offering opportunities during work hours or providing flexible alternatives. Supporting the emotional well-being of healthcare staff is critical, as their ability to care for patients is inherently linked to their own mental health [52].

### Limitations

The present study has some limitations. The main one was the low participation. The small sample size in this study reflects a significant limitation, primarily due to the nature of the research context, even though data saturation were achieved. It is a subject that can be emotionally stirring, and not all healthcare workers are prepared for it. On the other hand, cultural limitations in understanding perinatal loss and infant death influence the interpretation of results. Future research should consider strategies to encourage higher participation rates and explore diverse cultural perspectives on perinatal bereavement care [53]. Another limitation is the potential bias introduced by group dynamics during the focus group discussion, such as the influence of dominant voices, which may have affected the diversity of perspectives. While efforts were made to mitigate this through skilled moderation techniques, the inclusion of additional data sources, such as individual interviews, could provide further triangulation and strengthen the validity of the findings in future research.

## 5. Conclusions

The nursing staff is a fundamental pillar in the care of families who have suffered a perinatal loss. This task demands great personal and emotional involvement, which can be challenging under certain conditions. Beyond emotional commitment, it is essential to receive training in bereavement care to provide appropriate support that considers the families’ needs and demonstrates empathy at all times.

Additionally, the development of protocols should consider the experiences of the personnel who will implement them in clinical practice, since their experiences make the protocol closer to the reality of the cases.

Our findings revealed key barriers to effective bereavement care, including knowledge deficits among nursing staff, emotional strain caused by their involvement in care, and perceived inadequacies in the current protocol. Addressing these challenges through targeted training and protocol adjustments is essential for improving both the quality of care and the emotional well-being of healthcare professionals.

To improve the effectiveness of perinatal bereavement care, this study highlights several actionable recommendations:

- Reevaluate the use of the CuddleCot: Assess its effectiveness and explore alternative methods that may better support families, based on their preferences and feedback.
- Enhance family support options: Introduce early psychological interventions and continuous emotional support for families from the moment of diagnosis through the grieving process.
- Improve staff training: Incorporate simulation-based learning and culturally sensitive communication strategies into training programs to better equip staff for emotionally demanding situations.
- Optimize protocol communication: Ensure clear and accessible communication of the protocol to all staff members, possibly through interactive and practical training sessions rather than solely online modules.
- Adapt logistical procedures: Improve the process for transferring deceased infants to the mortuary, using more appropriate and sensitive methods to minimize emotional impact.

These recommendations are directly based on our findings, which revealed challenges such as the lack of formal training, emotional strain among nursing staff, and practical difficulties in implementing the perinatal bereavement protocol. Addressing these issues through targeted strategies will improve the quality of care provided to bereaved families and support healthcare professionals in managing emotionally demanding situations.

Moreover, involving nursing staff in the design and continuous evaluation of the protocol ensures that care practices remain realistic and aligned with both clinical needs and family expectations.

## Data Availability

The data presented in this study are available on request from the corresponding author. The data are not publicly available due to privacy and ethical restrictions.

## Supplementary Materials

The following supporting information can be downloaded at: https://www.mdpi.com/article/10.3390/healthcare13030242/s1, Table S1: Major and Minor Themes with Participant Quotations.

## Artificial Intelligence (AI)

Tools were used to assist in the refinement of language and content organization. All data analysis, interpretation of results, and final decisions regarding the manuscript were made solely by the authors.
