# Supplementary material for: Healing Through Humanized Care: Lessons from a Patient-Centered Perinatal Loss Protocol
Source: Healthcare (Basel). 2025 Jan 24;13(3):242. doi: 10.3390/healthcare13030242 (PMC11816378; doi:10.3390/healthcare13030242)
Supplement: Supplementary file 1 [file healthcare-13-00242-s001.zip › healthcare-3425334-supplementary.pdf]

Mayor and minor themes, and participants quotations.

Mayor themes	Minor themes		Quotations
Nursing staff	Strengths	Empathy	*"When a mother comes with her baby, I try to find out the baby's name… You address the baby by their name, and it gives the family dignity" "Since the mother was going to hold her baby, I tried to prepare everything as best as I could so that she wouldn't have that memory engraved in her mind" (P1)*
	Weaknesses	Lack of knowledge	*"It's a situation that you try to avoid entering because sometimes you don't know how to face or how to console the family. Well, at the beginning a bit more... Then you get the hang of it" (…) " I took a course on that, and I apply it a bit in these cases, but of course I don't have any other training. And I don't think my colleagues in here have any" (P1)*<br><br>*"I don't have any information, nor have we been given it here. I don't have much training either... I think that's what it feels like"(P2)*
		Lack of empathy	*"It was hard to work because you had to get into it, to empathise with her, to put yourself in her shoes. Not having been a mother myself, maybe I empathise less" (P3)*

			"Here everyone (the family) comes in to say goodbye to the child, so this is not fair for us" (P2, all assent)<br><br>*"For example, if a child dies at the age of 8 (...) and you are not allowed to keep him in your arms for 6 hours, I can't understand it (…) Because this mother, what bond does she have with this child if she has just seen him?" (P4)*
		Emotional imprint	*"I used to sleep and dream about the child (…) That day was not pleasant, when I got home, I saw the child, I saw the image of the child. You forget that, but it is true that it makes an impression (…) So when I see a situation like that with that child there, lifeless, it is a deep pain for me"(P3)*
		Rejection for self-protection	*"I found the experience the most unpleasant thing in the whole world." (P1)*<br><br>*"As I told you, for me is violent... I'm not convinced that the child stays here, I'm not convinced" (P3)*<br><br>*" Thank God, I have not received any of them in a while" (P4)*
The Perinatal Bereavement Protocol	Positive key points	Creating memories	*"Normally the midwives come up with the souvenir envelope, (...) a bracelet identifying the baby, the footprint, if they have put a hat on the baby... They leave it*

			there in a little envelope so that they can keep it as a remembrance. They are allowed to accompany her. I don't have so many measures for that, if they come in they don't crowd the room, but well, I allow different relatives to come in to accompany the mother above all" (P1)<br><br>" *As bad as it is... you can't even leave it on the 3rd floor (delivery ward), they're already there listening to other mothers with their babies crying, and obviously they're not going to take it up to the 4th floor (maternity ward)" (P4, all assent).*<br><br>*"It is true that when women are given information about the psychological support consultation, it helps them in the grieving process... (...) in my opinion, it is as if they feel a little relieved" (P2)*
	Negative key points	Not involving the staff in the development	*"A circuit (online training) was done, but I don't think that is the way to transmit information to a staff that takes care of this kind of patients" (P1)*<br><br>(Talking about face-to-face training) *"Well, if it gives the hours, yes, but as the house no longer gives hours..." (P1)*<br><br>"We haven't been given much training either... that's how we feel" (P2)

			"(...) she drew up his protocol, she passed it around like this from time to time, 'to see if you think something is missing or not'. But let's say one thing... as if I pass it on to you, but he says 'well, in the end it's the way I have it oriented'. Personally, I don't think that's the way to give perinatal bereavement training"(P3)<br><br>*"It was implemented with a lot of ignorance on the part of the staff of what needed to be done here. Someone would come in and say 'hey, have you asked her if she wants to use the milk to make earrings' (...) but we were completely unaware of that (...) they told us where the cot was and we didn't even have the key to get in". (P4)*<br><br>*"You are here alone at night and suddenly they call you and you don't know where you stand because you don't even have anyone to ask" (P4, all assent)*<br><br>*"The gynaecologists don't know where to call, nor do you" (P3)*
		The baby's physical appearance	*"It is true that the sensation... is that for me the sensation of the child's touch (...) I couldn't even look at him because even the touch was creepy, because it was a sensation of a body, like a mashed, soft body, your hands would sink into the little head...(...) I don't know, you'd have to ask the mother..." (P1)*

			" I think that the mother and father should be told what the child will look like, so that they too can get the idea that this is not a body like that of an adult person, which, when it dies, has its own consistency for a few hours. I think they should prepare the mother and the father so that they know how they are going to find their child so that they can keep it for 6 hours or say 'well, we'll see the baby 5 minutes'" (P2)
		Emotional bereavement for the family	*"I think that the families who have the baby here are more broken when they take the baby away, it's harder for them to finish pushing (...) when you have the baby for 6 hours and now they take it away, the rest of the afternoon and evening I see them as more broken, it's harder for them to carry on afterwards..."(P2, other participants assent).*<br><br>*"But now that the foetus is also here, that's negative (...) I imagine that the mother is already completely broken"(P3)*
		Protocol implementation – inadequate management	*"Very poorly assessed. In other words, the experience may be good, but here it was implemented a little too fast" (P4, others participants assent)*<br><br>*"Here you know what gynaecologists are like, "that the necropsy paper is missing", when not "that the transfer paper is missing", "that I am missing another one". And*

			you have that woman who has already done the relevant hours, (...) that bureaucracy is very badly managed, (...) Something is always missing afterwards"(P3)<br><br>*"The cradle of hugs is a fallacy, because the cradle of hugs is a metaphorical and poetic name that they wanted to give to something" (P2)*<br><br>*"When a woman comes with a baby that has died, it is passed from hand to hand by all the relatives. The tendency is to hold it and not to use it for anything." (P4, all assent)*<br><br>*"And then to close the whole circle, which the parents don't see, but I think it's very bad that you have everything set up, a super cool protocol, and for the transfer of a deceased child you bring the baby, which is a mammoth thing, to transfer a small child like that" (P1)*
What's next?	Bereavement protocol	Improvement strategies	*"Training to make it easier for you to interact with her, or to express yourself, or not to use phrases that might hurt (...) how to deal with bad news, how to empathise with a person who is suffering a perinatal bereavement" (P4)*<br><br>*"I would remove the cradle, I would put in the child transport" (P2, other participants assent).*

			"They should assess whether what they are projecting, that these women should be followed up, that these patients should be interviewed over time to see if being accompanied by their baby in the room for the hours that it was there has helped them, in their process of grief, of mourning, has helped them in any way (...) I would follow them up over time to see if it has helped them in any way". (P1)<br><br>*"I think that maybe more help in the form of a psychologist, from the first moment, not waiting for a week, but from the first moment a person who is supporting the couple, I think it is better than having the child there passing it from hand to hand" (P3, other participants assent).*

* P1: Participant 1; P2: Participant 2; P3: Participant 3; P4: Participant 4.
